# Phytotoxicity, Morphological, and Metabolic Effects of the Sesquiterpenoid Nerolidol on *Arabidopsis thaliana* Seedling Roots

**DOI:** 10.3390/plants9101347

**Published:** 2020-10-12

**Authors:** Marco Landi, Biswapriya Biswavas Misra, Antonella Muto, Leonardo Bruno, Fabrizio Araniti

**Affiliations:** 1 Department of Agriculture, Food and Environment, University of Pisa, 56126 Pisa, Italy; marco.landi@unipi.it; 2 Independent Researcher, Pine 211, Raintree Park Dwaraka Krishna, Namburu AP-522508, India; bbmisraccb@gmail.com; 3 Dipartimento di Biologia, Ecologia e Scienze della Terra (DiBEST), Università della Calabria, 87040 Arcavacata di Rende, CS, Italy; antonella.muto@unical.it (A.M.); leonardo.bruno@unical.it (L.B.); 4 Department AGRARIA, University Mediterranea of Reggio Calabria Località Feo di Vito, 89124 Reggio Calabria, RC, Italy

**Keywords:** phytotoxicity, herbicide, root morphology, sesquiterpene alcohol, metabolomics

## Abstract

Natural herbicides that are based on allelopathy of compounds, can offer effective alternatives to chemical herbicides towards sustainable agricultural practices. Nerolidol, a sesquiterpenoid alcohol synthesized by many plant families, was shown to be the most effective allelopathic compound in a preliminary screening performed with several other sesquiterpenoids. In the present study, *Arabidopsis thaliana* seedlings were treated for 14 d with various cis-nerolidol concentrations (0, 50, 100, 200, 400, and 800 µM) to investigate its effects on root growth and morphology. To probe the underlying changes in root metabolome, we conducted untargeted gas chromatography mass spectrometry (GC-MS) based metabolomics to find out the specificity or multi-target action of this sesquiterpenoid alcohol. Oxidative stress (measured as levels of H_2_O_2_ and malondialdehyde (MDA) by-product) and antioxidant enzyme activities, i.e., superoxide dismutase (SOD) and catalase (CAT) were also evaluated in the roots. Nerolidol showed an IC_50_ (120 µM), which can be considered low for natural products. Nerolidol caused alterations in root morphology, brought changes in auxin balance, induced changes in sugar, amino acid, and carboxylic acid profiles, and increased the levels of H_2_O_2_ and MDA in root tissues in a dose-dependent manner. Several metabolomic-scale changes induced by nerolidol support the multi-target action of nerolidol, which is a positive feature for a botanical herbicide. Though it warrants further mechanistic investigation, nerolidol is a promising compound for developing a new natural herbicide.

## 1. Introduction

Weeds are one of the major threats to global agroecosystems, as they affect both crop productivity and quality [1]. The use of synthetic herbicides that are easy to apply and are economically accessible to farmers, is one of the popular and effective methods of weed management [2]. Nevertheless, the excessive use of chemical herbicides has negatively influenced the ecological equilibrium and human health [3]. Moreover, it has been clearly demonstrated that the majority of the known herbicides target a single specific metabolic action site [4,5,6], which is the main factor resulting in a rapidly evolving resistance to these synthetic chemicals.

Herbicides with new mechanisms of action are extremely needed to counter this rapidly increasing evolution of herbicide resistance [7]. In addition, the attention of the public to possible hazardous effects of chemical herbicide to human health is continuously increasing, and new research activities are actively moving toward the search of naturally-derived herbicides, based on the allelopathic properties of some natural compounds [8,9,10].

Nowadays, it is of utmost importance for the use of a combination of agronomic, physical, mechanical, and chemical strategies for weed control in an Integrated Weed Management System (IWMS) [11,12] framework. In particular, the prospect of using secondary plant metabolites as natural herbicides, or as the backbone for herbicide discovery programs is becoming an effective alternative to the classic synthetic herbicides [13]. Natural compounds can effectively inhibit weed performances, act simultaneously on specific, and in most cases, multiple targets [14,15,16,17]. This ability to alter plant metabolism at different biochemical checkpoints increase the effectiveness of these compounds, but at the same time, it renders the identification of their main and most effective modes of action challenging [18]. Moreover, the chemical structure of natural products is generally more complex than synthetic herbicides, which means that they would not be easily obtained by traditional synthetic approaches based on massive chemical syntheses with the production of countless compounds whose biological activity is totally unknown [19].

Nerolidol (C_15_H_26_O; MW, 222.37 Da; IUPAC: 3,7,11-Trimethyl-1,6,10-dodecatrien-3-ol; also known as peruviol and penetrol), is a naturally occurring sesquiterpene alcohol found in the essential oils of diverse plants and flowers [20], and contributes to the fragrance/essence of the plants. This compound is largely studied for its multi-faceted pharmacological and biological activities [20,21,22] as well. Other compounds belonging to the sesquiterpenoid classes have shown a strong herbicidal activity [14,23]. In addition, nerolidol has already been mentioned as an important component of Asteraceae essential oil, whose natural herbicide activity was demonstrated [24]. Further, in our pilot studies, we have found that nerolidol resulted as a promising candidate among other sesquiterpenes (unpublished data) for allelopathy.

Many allelochemicals, as stress inducers in acceptor plants, cause morphological alterations, and reduce weed germination and vigor [17,23,25,26,27]. However, their effectiveness in germination and growth inhibition of the weeds is, in most cases, the result of more complex metabolic alteration induced in the affected plants [16,27]. It is, therefore, essential to understanding the biochemical mechanism of action of these naturally-derived compounds for a future application as either a whole molecule, as a co-formulant, or as a backbone for a derived herbicide.

In the present study, we focused on the effects of nerolidol on root morphology and root metabolome of *Arabidopsis thaliana* (L.) Heynh. Columbia-0 seedlings were treated for 14 d with a range of concentrations of this sesquiterpene alcohol. We also probed the effects of this compound on auxin balance to understand the underlying mechanism(s) responsible for the nerolidol-induced root alterations and the associated strong growth inhibition to elucidate the single- or multi-target activity of this potential allelochemical herbicide.

## 2. Materials and Methods

### 2.1. Root Bioassays in Arabidopsis thaliana Col-0

Cis-Nerolidol (Sigma-Aldrich, Milan, Italy, Cat. No. 72180-25ML) was firstly dissolved in 0.1% EtOH (ethanol, *v*/*v*) and was then diluted in deionized ddH_2_O to reach the final concentrations of: 0, 50, 100, 200, 400 and 800 µM. The same amount of EtOH was added to the control treatments (0 µM nerolidol). *Arabidopsis* seed sterilization and synchronization of the germination process were carried out as previously described by Araniti et al. [28]. To evaluate the phytotoxic effect of nerolidol on root morphology, seeds of *Arabidopsis* were germinated on Petri dishes (100 × 150 mm) containing agar medium (0.8% *w*/*v*), enriched with micro- and macronutrients (Murashige-Skoog basal salt mixture, Sigma-Aldrich, Milan, Italy, Cat. No. M5524-50L) and supplemented with 1% sucrose as carbon source. Then, petri plates were transferred to a growth chamber (21 ± 2 °C temperature and 75 mol·m^−2^ s^−1^ light intensity). Immediately after germination of five seedlings, for each replicate and treatment, seedlings were transferred on treated Plates prepared as previously described. After 14 d of treatments the whole root system was imaged by scanning (STD 1600, Régent Instruments Inc., Quebec, QC, Canada) and Primary Root Length (PRL), and Number of Lateral Roots (NLR) were measured using WinRhizo Pro system v. 2002a (Instruments Régent Inc., Quebec, QC, Canada), whereas Root Hair Density (RHD) and Root Hair Length (RHL) were analyzed using a stereo-microscope (Olympus SZX9, Italy) and the software Image Pro Plus v6 (Media Cybernetics, Rockville, MD, USA).

### 2.2. Quantification of Indole-3-Acetic Acid (IAA)

Quantification of indole-3-acetic acid (IAA) quantification was carried out following the method proposed by Rawlinson et al. [29] with some modifications. *Arabidopsis* roots were cut with a sharp blade below the hypocotyl, and were immediately frozen in liquid nitrogen, ground to a fine powder and were aliquoted. Further, weighed amounts of the powder (100 mg) per treatment and replicate were poured into 2 mL microcentrifuge tubes for extraction. To the samples, 20 µL of a 20 mg/mL solution of 3-indolepropionic acid (IPA) was added as internal standard for quantification and normalization purposes. Successively, 200 µL of NaOH (1% *w*/*v*), 147 µL of methanol (MeOH) and 34 µL of pyridine were added and the samples were vortexed for 40 s. To the extracted samples 20 µL of methyl chloroformate were added and samples were again vortexed for 30 s (and this step was repeated twice). To this extract, 400 µL of chloroform was added, samples were shaken for 10 s and 400 µL of a NaHCO_3_ solution (50 mM stock) was added. Samples were shaken again for 20 s and were immediately centrifuged (14,000 rpm) for 1 min. The organic lower phase was collected and dispensed into a new 2 mL centrifuge tube and the aqueous residues were eliminated using anhydrous Na_2_SO_4_. An aliquot (100 µL) of this organic phase was used for gas chromatography mass spectrometry (GC-MS) analysis. A parallel experiment was carried out using pure IAA (Sigma Aldrich, 20149, Milano, Italy, Cat. No. I3750-25G-A) as an external standard for retention time (RT) assignment and for the generation of a standard curve.

GC-MS analysis was carried out using a Thermo Fisher (Thermo Fisher Scientific, 20090 Rodano (MI), Italy) gas chromatograph apparatus (Trace 1310) equipped with a single quadrupole mass spectrometer (ISQ LT). The capillary column (MEGA-5MS 30 m × 0.25 mm × 0.25 µm) was connected to a 10 m long pre-column (MEGA S.r.l., 20025 Legnano (MI), Italy), and the carrier gas was helium with a flow rate of 1 mL/min. Injector and transfer line were set at 250 °C and 270 °C, respectively. Samples (3 µL) were injected with a 35 psi pressure pulse, which was held for 1 min. The following GC temperature program was used: Isocratic for 1 min. at 40 °C, from 40 °C to 320 °C with a rate of 20 °C min^−1^, then held isocratic for 2 min at 320 °C. The ion source was set to 200 °C and the solvent delay was 4.5 min. Mass spectra were recorded in electronic ionization (EI) mode at 70 eV, scanning at 50–400 m/z range to select appropriate EI mass fragments for each analyte. Then the MS was run in selected ion monitoring (SIM) using one quantifier (m/z) and two qualifiers (m/z) ions. In particular, for IAA-methyl ester the ions 189, 103, 77 were selected for quantification. IAA identification and quantification were performed by comparing the RT with the IAA external standard and the mass spectra in the National Institute Standard and Technology (NIST 2011) spectral library. The relative IAA quantification was carried out normalizing the IAA peak intensity with the intensity of the internal standard.

### 2.3. In-Situ Semi-Quantitative Detection of H_2_O_2_

The in-situ semi-quantitative detection of H_2_O_2_ was carried out as previously described by Araniti et al. [30] with some modifications. Root tips of the seedlings were treated for 14 d with nerolidol 120 μM were freshly cut, transferred to acidified water (pH 3.8) containing 3,3-diaminobenzidine (DAB) and were infiltrated in vacuum conditions for 5 min. After infiltration, roots were incubated in dark for 2 h. Successively, root tips were illuminated until the appearance of the characteristic reddish-brown color. The experiment was repeated ten times (*n* = 10).

### 2.4. Quantification of Total Soluble Proteins, Lipid Peroxidation (Malondialdehyde, MDA), Superoxide Dismutase (SOD) and Catalase (CAT) Activities

The total soluble protein content of *A. thaliana* roots was determined using Bradford [31] method, using 100 mg of fresh weight root material per replicate and treatment. Bovine serum albumin was used as standard and the soluble protein content was expressed as µg/g of dry weight. Dry weight estimation, carried out on five independent samples per treatment, was recorded by drying 1 g of treated and untreated fresh plant materials at 60 °C until a constant weight was reached. Enzymes involved in antioxidant system, such as superoxide dismutase (SOD), catalase (CAT) and lipid peroxidation (using malondialdehyde, MDA) were quantified by colorimetric methods using commercial kits from Sigma Aldrich (MDA, MAK085-1KT; SOD, 19160-1KT-F; CAT, CAT100-1KT) and samples were analysed according to manufacturer’s directions. Protein estimation was carried out in quadruplicates (*n* = 4), whereas MDA, SOD and CAT were measured ten times apiece (*n* = 10).

### 2.5. Metabolite Extraction and Derivatization for GC-MS Based Untargeted Metabolomics

Metabolite extraction and derivatization were carried out as previously described by Araniti et al. [27] using ribitol (0.2 mg/mL stock in ddH_2_O) as an internal quantitative standard. The derivatized extracts were injected into a TG-5MS capillary column (30 m × 0.25 mm × 0.25 µm) (Thermo Fisher Scientific, Waltham, MA, USA) using a gas chromatograph apparatus equipped with a single quadrupole mass spectrometer previously described. Injector and source were set at 250 °C and 260 °C temperature, respectively. Samples (1 μL) was injected in splitless mode with a helium flow of 1 mL/min as a carrier gas, using the following programmed temperature: Isothermal 5 min at 70 °C followed by a 5 °C/ min ramp to 350 °C and a final 5 min heating at 330 °C. Mass spectra were recorded in electronic impact (EI) mode at 70 eV, scanning at 40–600 m/z range, scan time 0.2 s. Mass spectrometric solvent delay was settled as 9 min. n-Alkane standards (C_10_–C_40_ all even), blank solvents were injected at scheduled intervals for instrumental performance, tentative identification, and monitoring of shifts in retention indices (RIs).

### 2.6. Analyses of GC-MS Metabolomics Data

Raw GC-MS data were then analyzed using the open source software MS-DIAL ver. 4.24 coupled with open source publicly available and commercial EI spectra libraries (NIST 2011; GOLM database, MassBank; Mass Bank of North America etc.) [32,33,34]. MS-DIAL parameters were as follows: Average peak width of 20 scan and minimum peak height of 1000 amplitudes was applied for peak detection, and sigma window value of 0.5, EI spectra cut-off of 10 amplitudes was implemented for deconvolution. For identification setting, the retention time tolerance was set to 0.5 min, the m/z tolerance was set to 0.5 Da, the EI similarity cut-off was 70%, and the identification score cut-off was 70%. In the alignment parameters setting process, the retention time tolerance was 0.075 min, and EI similarity tolerance of 70%. Metabolite annotation was carried comparing the retention index and the spectra similarity of the samples with those of the libraries, following the Metabolomics Standards Initiative (MSI) levels of the International Metabolomics Society: Reported annotations were considered level 2 (putative annotation based on spectral library similarity) or level 3 (putatively characterized compound class based on spectral similarity to known compounds of a chemical class as suggested elsewhere [35]. Only compounds with a S/N ratio higher than 10 were considered for annotation.

### 2.7. Statistical Analyses

#### 2.7.1. Statistical Assessment of Morpho-Physiological Parameters

All the experiments were carried out in a completely randomized design with different replications depending on the parameter evaluated. Dose-response curves and their parameters (PRL, NLR, RHD and RHL) were analyzed through one-way ANOVA using least significant difference (LSD) test (*p* ≤ 0.05) as post hoc test. The experiments were replicated 5 times (*n* = 5). Data on IAA (*n* = 4), total proteins content (*n* = 4), MDA (*n* = 10), SOD (*n* = 10) and CAT (*n* = 10) were analyzed through the Student test (*p* ≤ 0.05).

The PRL responses to different doses of nerolidol were analyzed through a nonlinear regression model using a log-logistic equation, largely employed in phytotoxicity screenings [36] to estimate the IC_50_ parameter, the dose required to reduce 50% of the total response (i.e., root growth).

#### 2.7.2. Metabolomic Data Analysis

The GC-MS metabolomics data were analyzed using the web-based open source statistical tool MetaboAnalyst ver. 4.0 [37]. The metabolite abundances were checked for integrity, and missing values were replaced by a small positive value (the half of the minimum positive number detected in the data) and features with > 50% missing values were removed. Data were successively normalized by a reference metabolite (ribitol), transformed through “log2 normalization” and were scaled by Auto-Scaling. The data were then subjected to unsupervised multivariate Principal Component Analysis (PCA) and the metabolite variations are presented as a heatmap. Data were then analyzed through the univariate analysis to yield volcano plots that demonstrated the significantly differential metabolites with a FC higher than 1 and a *p* ≤ 0.05 (Student’s *t*-test). Volcano plot was created using Multiplot (v2) (https://www.genepattern.org/modules/docs/Multiplot/2) tool by plotting-log10 (*p*-values) as Y-axis and lo2 (fold changes) as X-axis as a scatter pot within GenePattern functionalities [38].

Pathway analysis was carried out using the *Pathway Analysis* function in the MetaboAnalyst webserver, which combines both enrichment and topology analysis that evaluates the possible biological impacts based on the perturbed pathways. Only pathways with an impact higher than 0.15 were considered as significantly altered in this study.

### 2.8. Raw Data Sharing

The raw datasets and the metadata associated with the GC-MS-based metabolomics efforts are deposited at the Mendeley database (DOI: 10.17632/v33fjk4fmm.1) and are freely available for download from 26 November 2020 here: https://data.mendeley.com/datasets/v33fjk4fmm/1.

## 3. Results

### 3.1. Dose-Response Curves to Measure the Effects of Nerolidol on Root Morphology

We observed that nerolidol treatments strongly affected the root morphology of *A. thaliana* seedlings and significantly altered the root tip organization more specifically at the higher concentrations. In particular, nerolidol significantly inhibited the Primary Root Length (PRL) of the treated seedlings.

**Figure 1:**
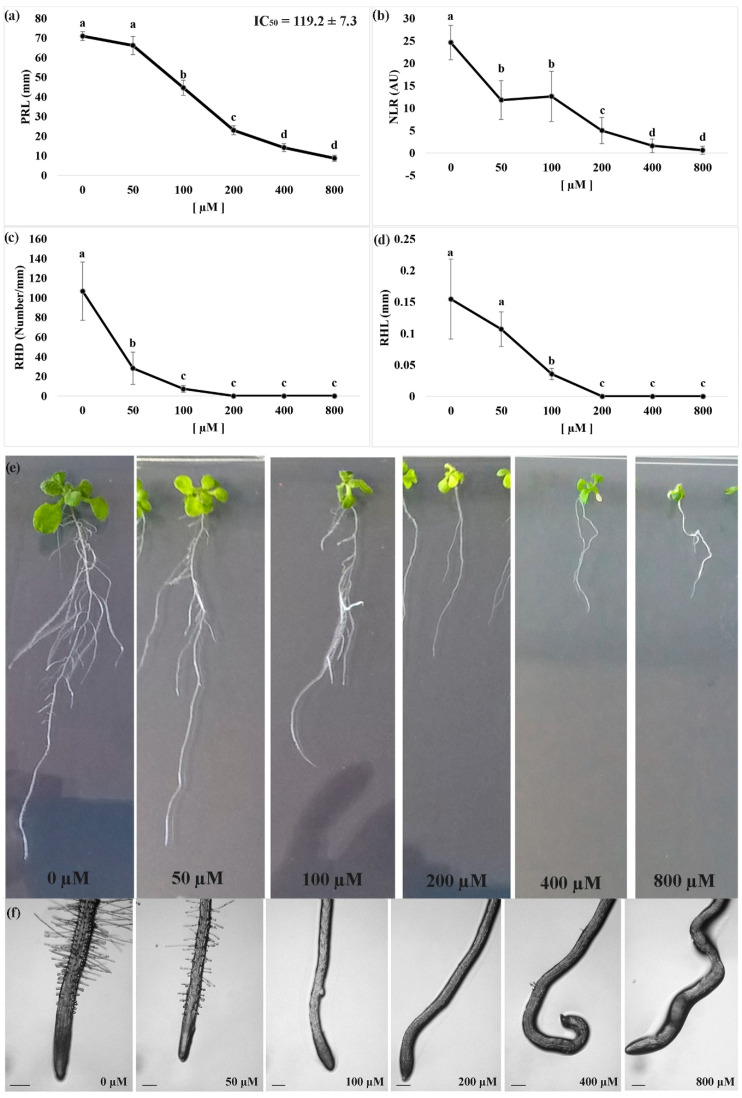
Dose response curve of *Arabidopsis* seedlings treated with different doses of nerolidol (0 µM–800 µM). (**a**) Primary root length (PRL); (**b**) number of lateral roots (NLR); (**c**) root hair density (RHD); (**d**) root hair length (RHL); (**e**) effects of different doses of nerolidol on root morphology, and (**f**) root tips of *Arabidopsis* seedlings treated for 14 d with nerolidol. Data are expressed as mean ± SD. Different letters indicate significant differences observed among treatments at *p* ≤ 0.05 (LSD test). *n* = 5 for PRL and NLR. *n* = 20 for RHD. Root tips images were photographed using an Olympus stereo microscope, with a 40× magnification. Scale bars in Figure 1f = 200 µm.

The lowest concentration of nerolidol administered did not significantly affect the PRL, whereas a 100 µM nerolidol treatment induced a growth reduction by ~40% that reached to a 70% inhibition at 200 µM, when compared to control (i.e., mock EtOH treated) seedlings (Figure 1a). Finally, the highest concentrations of nerolidol assayed (400 µM and 800 µM) induced an 80–90% of inhibition.

Observations on the Number of Lateral Roots (NLR) pointed out a similar trend of dose-dependent inhibition. As shown in Figure 1b, the lowest concentrations assayed (50 µM and 100 µM) similarly affected the NLR parameter inducing a ~50% inhibition when compared to control seedlings, for this parameter, whereas nerolidol concentration of 200 µM induced 80% inhibition (Figure 1b). The highest concentration of nerolidol applied induced NLR reduction higher than 90%.

Finally, both Root Hair Density (RHD) and Root Hair Length (RHL) parameters were observed to be extremely sensitive to nerolidol. Observation of RHD pointed out a 70% reduction at the lowest concentration (50 µM) and reached inhibition values higher than 90% at higher concentrations, whereas RHL pointed out an 80% of inhibition at a concentration of 100 µM and a complete inhibition at higher concentrations (Figure 1c).

The nonlinear regression fit of the PRL raw data quantifies the IC_50_ parameter (dose required to induce a 50% of inhibition of the studied parameter). In particular, the IC_50_ value, which has been successively used as a key concentration for the metabolomic experiments, corresponding to ~120 µM.

**Figure 2:**
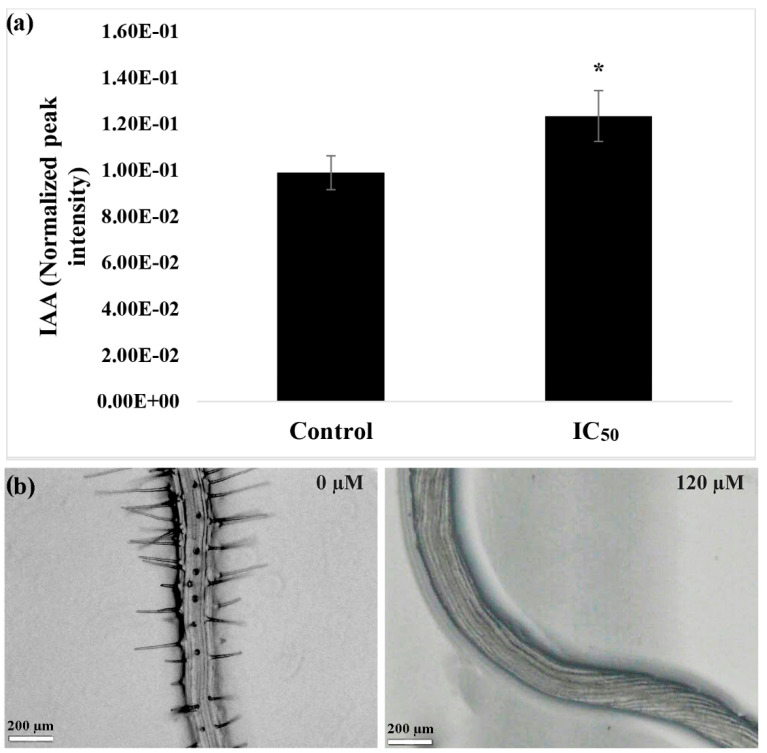
(**a**) The concentration of indole-3-acetic acid (IAA) in the roots of *A. thaliana* seedlings in control and nerolidol-treated seedlings at IC_50_ concentration (120 µM). (**b**) The bottom panels demonstrate morphological features of untreated roots, and IC_50_ concentration of nerolidol (120 µM) treated seedling roots. Note the anisotropic growth of the nerolidol-treated roots. Data are expressed as mean ± SD; * = *p* ≤ 0.05, *t*-test (*p* ≤ 0.05).

The bioassay of the IC_50_ concentration pointed out a significant disorganization of the primary root, which was characterized by corkscrew shape (Figure 1d,e). This phenomenon (Figure 2b) was already observed during the dose-response curve, but was becoming evident at concentrations ≥ 200 µM and less evident at 100 µM (data not shown).

### 3.2. IAA Quantification and Morphological Malformations in Roots

As reported in Figure 2a, the quantification of auxin in plants treated with nerolidol indicated an accumulation of auxin at 25% higher than in control. Moreover, as could be observed from the images reported in the bottom panel of Figure 2b, the maturation zone of the root of the treated plants is devoid of root hairs and is characterized by an anisotropic growth of the root that resembled a corkscrew shape.

### 3.3. In-Situ Semi-Quantitative Detection of H_2_O_2_

**Figure 3:**
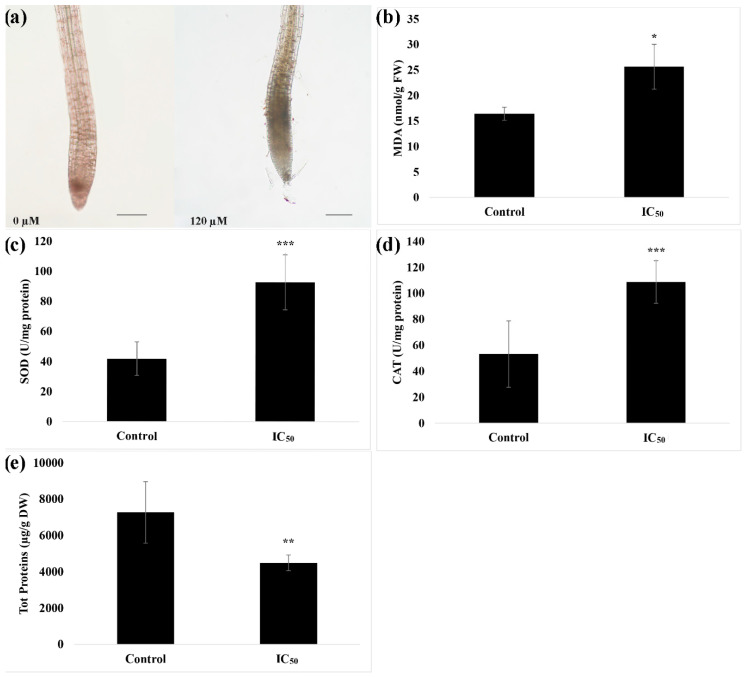
Effects of nerolidol at the IC_50_ concentration bioassayed for several biochemical parameters of *A. thaliana* roots. (**a**) *In-situ* semi-quantitative detection of H_2_O_2_; (**b**) lipid peroxidation (MDA) levels; (**c**) superoxide dismutase (SOD) activity; (**d**) catalase (CAT) activity; (**e**) total soluble protein content. Data are expressed as mean ± SD; * = *p* ≤ 0.05; ** = *p* ≤ 0.01; *** = *p* ≤ 0.001, *t*-test (*p* ≤ 0.05). Root tips images were photographed using an Olympus stereo microscope with a 40× magnification. Scale bars in Figure 3a = 200 µm.

The in-situ detection of H_2_O_2_ pointed out an accumulation of this reactive oxygen species (ROS) in the root meristem of seedlings treated with nerolidol (Figure 3a). This accumulation was accompanied by an increase of both ROS scavenging enzymes SOD (~1.2 folds higher than control) (Figure 3b), an increase of lipid peroxidation (~0.6 fold higher than control) (Figure 3c), CAT (~1 fold higher than control) (Figure 3d) and a significant concomitant reduction of total soluble protein content (~0.4 fold lower than control) (Figure 3e).

### 3.4. Metabolome-Wide Changes Induced by Nerolidol in A. thaliana Roots

To get more insights into the system-wide metabolic changes in response to an IC_50_ dose of nerolidol, a GC-MS-based untargeted metabolomic analysis was carried out that mainly captures the polar metabolites of the primary metabolism of plant tissues. Annotated metabolites across the two sample groups and treatments revealed 62 shared metabolites, belonging to the class of the amino acids, organic acids, sugars, sugar alcohols, polyamines, among others (Table S1).

**Figure 4:**
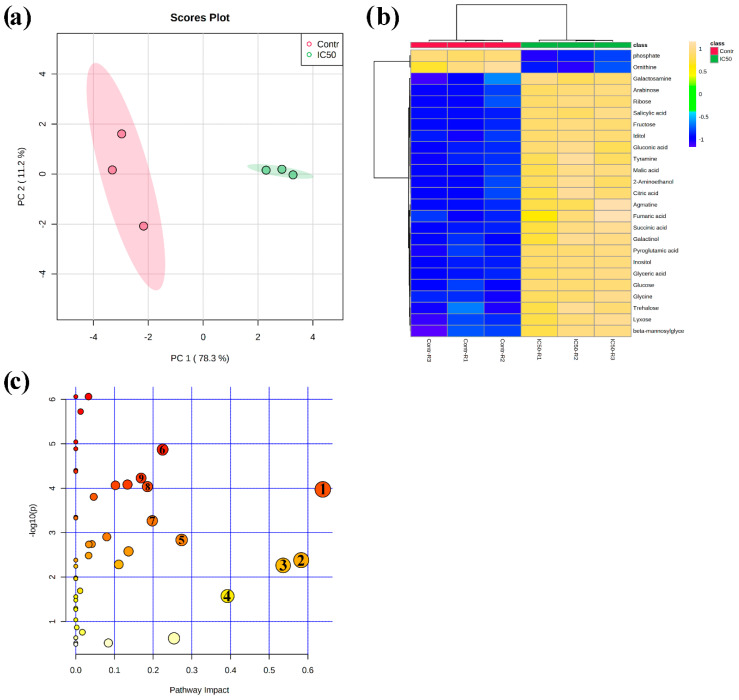
(**a**) Principal component analysis (PCA) score plot for control plants (red dots) and plants treated for 14 d with nerolidol 120 µM (green dots); (**b**) overlay heat map of the top 25 metabolites profiles (selected by *t*-test with *p* ≤ 0.05) in plants treated for 14 d with nerolidol 120 µM (IC_50_-R1–IC_50_-R3, replicates of the treated samples) in comparison with control plants (Contr-R1–Contr-R3, replicates of the controls). Each square represents the effect of the treatment on the relative abundance of every metabolite using a false-color scale. Colors yellow and blue indicate relative metabolite abundances, increased and decreased, respectively; (**c**) metabolomic pathway analysis showing the *p*-values from the pathway enrichment analysis and pathway impact values from the pathway topology analysis, numbered circles represent the pathways most-affected by the treatment (data used to build up the pathways graph are reported in Table 2). Full data are reported in Table S1 (raw data and PCA) and Table S3 (pathway analysis) (*n* = 3).

An unsupervised multivariate PCA score plot reported in Figure 4a demonstrates clear discrimination between control and IC_50_-nerolidol treated seedling root samples. Discrimination of the sample groups (120 µM nerolidol treatment and control plant roots) was achieved using the first two principal components (PCs) PC1 vs. PC2, which explained a total variance of 89.5%. In particular, PC1 explained 78.3% of the total variance, whereas PC2 explained 11.2% of the variance. The analysis of the PCA loading plots, which highlight the features strongly involved in groups separation, pointed out that the PC1 was mainly dominated by γ-aminobutyric acid (GABA), asparagine, urea, and threonine, whereas the PC2 was dominated by glycerol, urea, threonine, and 3-aminopropionitrile (Table S1).

**Figure 5:**
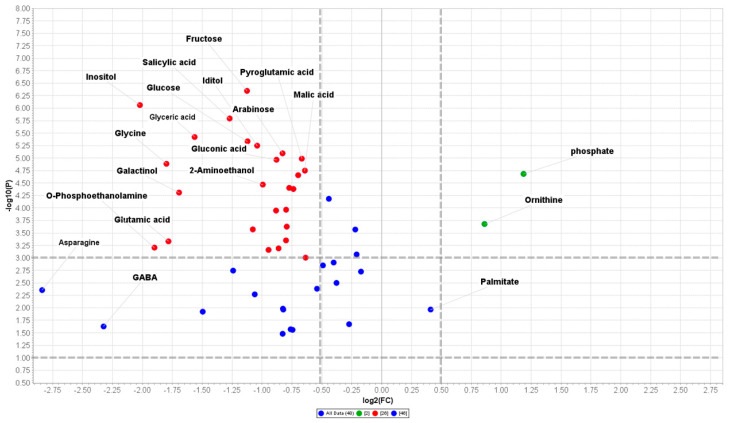
Volcano plot (FC = 1 and *p* value = 0.05) built on the metabolites annotated in 14 d nerolidol-treated roots of *Arabidopsis thaliana*. Full data are reported in Table 1 and Table S2. (*n* = 3).

A quick overview of the changes induced by nerolidol treatment was observed in the hierarchical clustering analysis (HCA) output as a heat map that further confirmed the clear separation between control and treated samples (Figure 4b). In fact, it can be noted that the HCA built using the Euclidean distance measure, and the ward algorithm pointed out the presence of two clearly separated groups, shown in Figure 4b, further confirming the PCA model. When using a fold change (FC) cut-off of 1 and a *p* value of 0.05, we presented a volcano plot that pointed out that 48 of the 62 annotated metabolites belonging to the biochemical classes of amino acids, polyamine, organic acids, and sugars, among others, were significantly altered by nerolidol treatment (Table 1). As shown in Figure 5 and Table 1, excluding metabolites, such as phosphoric acid, ornithine, and palmitic acid, all the metabolites altered by the nerolidol treatment were mostly significantly upregulated (full results of data analysis are reported in Table S2).

**Table 1:** The gas chromatography mass spectrometry (GC-MS) based metabolomic data obtained from *Arabidopsis* roots treated for 14 d with nerolidol concentration of 120 µM, showing significantly changed metabolites [cut offs; fold change (FC) ≥ 1 and a *p* value ≤ 0.05]. Full data are reported in Table S2 (*n* = 3).

Metabolites	FC: Control/IC_50_	*p*-Value (*t*-Test)	Biochemical Classes
Alanine	0.56342	0.033117	**Amino acids**
Allothreonine	0.5902	0.026942	
Asparagine	0.1398	0.004414	
GABA	0.19963	0.023587	
Glutamic acid	0.29068	0.000466	
Glycine	0.28728	1.30 × 10^−5^	
Leucine	0.59679	0.027607	
Serine	0.35456	0.011955	
Cadaverine	0.56438	0.010415	**Polyamines**
Ornithine	1.8154	0.000209	
N-acetylornithine	0.88723	0.001882	
Putrescine	0.56552	0.010855	
Tyramine	0.59957	4.16 × 10^−5^	**Amine**
Agmatine	0.54233	0.000113	
O-Phosphoethanolamine	0.26807	0.000623	
Citric acid	0.7364	6.54 × 10^−5^	**Organic acids**
Fumaric acid	0.57442	0.000446	
Gluconic acid	0.54372	1.08 × 10^−5^	
Glyceric acid	0.33833	3.78 × 10^−6^	
Malic acid	0.64095	1.78 × 10^−5^	
Succinic acid	0.58541	3.95 × 10^−5^	
Threonic acid	0.7115	0.001414	
Arabinose	0.5631	8.03 × 10^−6^	**Sugars**
Fructose	0.45831	4.50 × 10^−7^	
Glucose	0.45976	4.62 × 10^−6^	
Lyxose	0.57485	0.000108	
Maltose	0.55043	0.000644	
Ribose	0.61642	2.20 × 10^−5^	
Trehalose	0.47397	0.000268	
Glucose 6-phosphate	0.4793	0.005374	
Galactinol	0.30915	4.91 × 10^−5^	**Sugar alcohols**
Iditol	0.48601	5.64 × 10^−6^	
Inositol	0.24637	8.68 × 10^−7^	
Maltitol	0.64358	0.000988	
meso-Erythritol	0.82828	0.021309	
3-Hydroxy-3-Methylglutaric acid	0.51926	0.000691	**Organic acids**
6-Aminohexanoic Acid	0.76983	0.003172	
beta-Mannosylglycerate	0.57697	0.000237	
Dehydroascorbic acid	0.86524	0.000852	
Palmitate	1.3282	0.010824	
Pyroglutamic acid	0.6296	1.03 × 10^−5^	
Phosphoric acid	2.2766	2.08 × 10^−5^	
Salicylic acid	0.4143	1.61 × 10^−6^	
Shikimic acid	0.75721	0.001238	
Sinapinic acid	0.42289	0.001801	
2-Aminoethanol	0.50229	3.40 × 10^−5^	**Miscellaneous**
Galactosamine	0.85845	0.00027	
Glycero3-galactoside	0.68749	0.00415	

Finally, KEGG-based metabolic pathway analysis revealed that 21 pathways were significantly impacted by nerolidol treatment (at IC_50_ dose). However, only nine KEGG pathways were characterized by a pathway impact score higher than 0.15, and the most affected pathways were starch and sucrose metabolism, alanine, aspartate, and glutamate metabolism, and glycine, serine, and threonine metabolism, which were all upregulated and characterized by an impact higher than 0.5 (Table 2) (complete results of data analysis are reported in Table S2).

**Table 2:** KEGG-based metabolomic pathway enrichment and topology analysis for GC-MS derived metabolomics data, obtained from *Arabidopsis* roots treated with nerolidol 120 µM for 14 d. Metabolites were analyzed through the MetaboAnalyst tool for KEGG-based “*Pathway Analysis*”. Full data are reported in Table S3. *n* = 3.

Pathways	Total Compounds	Coverage	−log10(*p*)	FDR	Pathway Impact
Starch and sucrose metabolism (**1**)	22	5	3.976	0.000406	0.63853
Alanine aspartate and glutamate metabolism (**2**)	22	7	2.3819	0.007982	0.58274
Glycine serine and threonine metabolism (**3**)	33	6	2.2637	0.00973	0.53598
Arginine biosynthesis (**4**)	18	6	1.5727	0.037307	0.39224
Arginine and proline metabolism (**5**)	34	4	2.8369	0.003639	0.27366
Glyoxylate and dicarboxylate metabolism (**6**)	29	7	4.8696	0.000113	0.22451
Galactose metabolism (**7**)	27	6	3.2689	0.001584	0.1978
Citrate cycle (TCA cycle) (**8**)	20	3	4.0384	0.000381	0.18531
Tyrosine metabolism (**9**)	16	2	4.2311	0.000326	0.16892
Butanoate metabolism	17	3	2.5801	0.005717	0.13636
Glutathione metabolism	26	4	4.0848	0.000381	0.13362
Aminoacyl-tRNA biosynthesis	46	8	2.285	0.009608	0.11111
Inositol phosphate metabolism	28	2	4.0674	0.000381	0.10251
Phenylalanine tyrosine and tryptophan biosynthesis	22	1	2.9072	0.003258	0.08008
Glycerophospholipid metabolism	37	3	3.0159	0.002678	0.06525
Phenylpropanoid biosynthesis	46	1	2.7444	0.004173	0.0416
Sphingolipid metabolism	17	2	2.7361	0.004173	0.03365
Sulphur metabolism	15	2	2.485	0.006819	0.03315
Phosphatidylinositol signalling system	26	1	6.0615	2.17 × 10^−5^	0.03285
Amino sugar and nucleotide sugar metabolism	50	2	5.7243	3.14 × 10^−5^	0.0125
Fatty acid biosynthesis	56	3	1.6915	0.02992	0.01123

**Total Compounds**: The total number of compounds in a given pathway; **Coverage**: The actually matched number from the uploaded data; **−Log_10_(*p*)**: Logarithmic transformed *p* value (*p* value calculated through the enrichment analysis); **FDR**: *p*-value corrected through the False Discovery Rate (5%). **Impact**: The pathway impact value calculated from pathway topology analysis. (Numbers in parenthesis, 1–9 represent the pathways, shown in Figure 4).

## 4. Discussion

In the present study, we bioassayed the in vitro phytotoxicity of a sesquiterpenoid alcohol nerolidol in the model species *A. thaliana* that is sensitive to phytotoxins. We used a wide range of assay concentrations to identify the main target of the compound and to determine the key concentration (IC_50_) for the successive experiments aimed at identifying the mode of action of the molecule. Results indicate that nerolidol affected root growth and development in a dose-dependent manner. In fact, nerolidol at the highest concentration assayed, strongly altered the root morphology of *A. thaliana*, causing pronounced effects not only on morphology, but also to the anatomical structure of the roots that showed increased deformities upon treatment. In particular, at increasing doses, nerolidol inhibited the growth of the primary root, reduced the lateral root numbers, as well as root hair density and length. In addition, the treated seedlings activated several biochemical mechanisms involved in stress resistance, such as the elevation in the first line of defense against oxidative stress (i.e., increased SOD and CAT enzyme activities) and accumulation of central metabolites (sugars, amino acids, and polyols among others) that act as osmoprotectants.

### 4.1. Nerolidol Induced Root Morphological Alterations in a Dose-Dependent Manner That Involves IAA and ROS

Upon evaluation of the IC_50_ (defined as the dose necessary to reduce/ inhibit an evaluated parameter by 50%, i.e., root growth here), revealed that nerolidol reduced primary root growth at a dose of 120 µM, which is considered a relatively low concentration for a natural molecule. The use of IC_50_ concentration as a key dose for the successive experiments is of pivotal importance, since higher nerolidol concentrations completely deformed the roots and severely impacted the seedling growth, thereby rendering the treatment challenging for finding potential metabolic targets of the molecule. Similar alterations on root morphology and anatomy were observed with several phytochemicals (such as the sesquiterpene hydrocarbon farnesene and the polyphenol rosmarinic acid), and synthetic phytotoxins (such as synthetic coumarins and benzofurans, among others), as reported in previous studies [14,16,39,40].

Interestingly, the roots of nerolidol treated seedlings, at both IC_50_ and higher concentrations, exhibited anatomical alterations of the root in the maturation zone. In particular, the roots developed a corkscrew shape with no fixed orientation. A similar phenomenon, known as handedness, was previously observed in *Arabidopsis* treated with farnesene [14,28], with the difference that in the case of seedlings treated with farnesene, it showed a fixed direction (left-handedness) and was observed throughout the root.

Previous experiments demonstrated that handedness, as well as root morphological alterations, could be attributed to the ability of natural compounds that alter IAA concentration and distribution [15,41]. For example, it was demonstrated that farnesene at IC_50_ concentration induced the accumulation of auxin [14,15], as observed in our case. Further, farnesene induced an alteration in its transport to the root meristem, thereby inhibiting several efflux carriers, which eventually result in alteration of microtubule organization and the successive handedness phenomenon [14,15].

In fact, Li et al. [42] observed that with the allelochemical umbelliferone, IAA accumulation could mediate F-actin disruption, thus inducing strong malformations in the root tip. Furthermore, Romero-Puertas et al. [43] observed that 2,4-dichlorophenoxyacetic acid (2, 4-D, an IAA analog) caused root growth inhibition accompanied by H_2_O_2_ overproduction. In addition, Liptáková et al. [44] observed that an exogenous application of IAA induced oxidative stress and malformations in barley roots. This suggests that accumulation of IAA observed in nerolidol treated roots could have induced, as also observed during our experiments, the formation of ROS, which could have contributed to altered root morphology, as also previously demonstrated by other investigators [45,46].

In fact, as previously demonstrated by Dunand et al. [46], elevated accumulation of both H_2_O_2_ and superoxide reduced root length and root meristem size, as well as affected root hair formation and growth. Similarly, nerolidol-induced root alterations were also observed by Pasternak et al. [47] in *Arabidopsis*, after a short treatment with the known ROS inducer, alloxan. In our study, plants treated with nerolidol in-situ staining for H_2_O_2_ confirmed an increased accumulation of H_2_O_2_ in the treated roots. Among other consequences of ROS burst, damages to proteins and lipids are well known [47]. Membrane peroxidation driven by ROS accumulation could result in the formation of lipid oxidation products and protein degradation [48,49], as also observed in our study for nerolidol-treated plants. Moreover, protein degradation during biotic and abiotic stress exposure could also be a strategy adopted by plants to increase the osmoprotective amino acids [50,51]. Besides, H_2_O_2_ accumulation was accompanied by a stimulation of the two ROS scavenger enzymes, SOD, and CAT, regarded as the first line of defense mechanism in protecting plants from oxidative stress [52,53]. Our study results suggest that nerolidol-treated seedlings activated defense mechanisms to cope with the stress, although the high intensity of the brownish color in DAB-stained roots suggests that the ROS scavengers enzymes were not sufficient in scavenging all the generated H_2_O_2_ that was accumulating in the root tips.

Since auxin biosynthesis and signaling are tightly interconnected with the total hexose content [54,55], we decided to use an untargeted GC-MS based metabolomics approach, which monitors the changes of products of central carbon (primary) metabolism, i.e., carbohydrate levels in nerolidol-treated plants and to monitor the differential accumulation of several metabolites pivotal for plant growth, development, and defense against stress.

### 4.2. Nerolidol Regulated Primary Metabolic Pathways to Divert Energy for Stress Tolerance

Our untargeted GC-MS metabolomics data revealed system-wide changes in metabolism upon nerolidol treatment in *Arabidopsis* roots when compared to control seedlings. We envisioned that other than the ROS scavengers, there must be other routes using which the plants would be enduring an allelochemical induced stress. In fact, the activation of metabolic pathways aimed at elevating the production of osmoprotectants plays an important role in the protection against oxidative stress [56]. As observed in our unbiased metabolomics experiments followed by pathway analysis, nerolidol-treated seedlings were characterized by the activation and upregulation of several known pathways involved in stress response. Among them, several pathways involved in carbohydrate, amino acid metabolism, glyoxylate and dicarboxylate metabolism, as well as TCA cycle, were significantly impacted by the treatment with nerolidol. A volcano plot analysis performed with the relative abundance data of the metabolites affected in the roots of nerolidol-treated compared to control seedlings highlighted a higher accumulation of several amino acids (GABA, asparagine, etc.) [57], polyamines (cadaverine, putrescine, and ornithine) [58], simple carbohydrates (fructose, glucose, maltose, trehalose, and galactinol) [59,60], and organic acids (citric, malic, and fumaric acids) [61]-pathways. These aforementioned classes of organic compounds are well-known to be involved in plant defense against oxidative stress, generally referred to as compatible osmolytes, constitute an adaptive mechanism used by plants to cope with abiotic stresses, such as salinity, water deficit, heat, or cold [62,63,64].

For example, quaternary ammonium compounds, amino acids (such as asparagine and GABA), and polyols (such as galactinol), were all significantly elicitated by nerolidol treatment and possibly played a pivotal role in the recovery of plants from stress [65,66,67,68]. Notably, remarkable increases in GABA concentration occurs in plants in response to both abiotic (e.g., extreme temperatures, dehydration, salinity), as well as abiotic factors (e.g., mechanical damage, viral, and bacterial infections) [69,70]. Though GABA was discovered in plants over half a century ago [71], and studies on its roles as a primary metabolite have been well documented, evidence of potential mechanisms by which GABA acts as a signaling molecule in plants has only recently been reported (for a recent review see [72]). Therefore, the significant increases in GABA concentrations observed in our nerolidol-treated plants allow us to propose a signaling role for this compound, especially in coordination with other key signaling molecules (e.g., ROS) and phytohormones [73,74]. In addition, several studies, including ours, indicate that plants subjected to both biotic and abiotic stress, tend to reduce their total soluble protein content and/ or facilitate rapid protein degradation in favor of amino acids (known osmoprotectants) accumulation, to counteract the deleterious effects of ROS accumulation [75,76].

Among the pathways significantly impacted by nerolidol treatment, the starch and sucrose metabolism were the most dysregulated. Further, it is largely well studied that sugars, under stress conditions, act as both osmoprotectants and signaling molecules. For example, it has been demonstrated that in *Arabidopsis*, simple carbohydrates, such as glucose, functions as a hormone-like signaling molecule modulating plant growth and development, by interacting with phytohormone auxin polar transport and biosynthesis [77]. In addition, it has been reported that glucose metabolism and auxin signaling pathways interact to regulate root growth and development in *A. thaliana* seedling roots [53]. This study highlighted that glucose, which is significantly stimulated by nerolidol treatment in our study, interacts with auxin signaling, biosynthesis, and transport machinery to control root growth and its architecture under changing nutrient conditions [53]. In rice plants exposed to heavy metal stress, Mishra and Dubey [78] observed an increase in the activities of enzymes involved in sucrose degradation, followed by an accumulation of soluble sugars that serve as osmolytes to maintain cellular homeostasis, and regulate ROS balance in the cell. Moreover, simple carbohydrates, such as glucose, fructose, and trehalose, that showed significantly increased accumulation in nerolidol-treated roots, are known to be important for their antioxidant and osmoprotective function during plant stress [79,80].

In *A. thaliana*, trehalose is known to alleviate oxidative stress, ionic imbalance, and cell death induced by salinity [81]. Moreover, both hexoses and trehalose participate in responses to both abiotic and biotic stresses, since they act either as sources of carbon or as signaling molecules, activating a number of pathways connected to mitochondrial respiration [79]. Further, soluble sugars that are used by plants during stress as a source of carbon for plant maintenance [82,83] and several tricarboxylic acids, such as malic and fumaric acid, could be used by plants during stress conditions to generate energy and carbon skeletons for the production of other metabolites [84]. Furthermore, it has been proven that fumaric acid could be useful in the maintenance of cellular turgor pressure and pH, as well as serving as an accessible transient storage form of fixed carbon [85]. Another TCA cycle metabolite, citric acid ameliorates plant performance during stress enabling plant protection against ROS insults [86]. In addition, nerolidol-treated plants demonstrated higher accumulation of several polyamines, such as cadaverine, ornithine, and putrescine that are proven osmoprotectants that accumulate during both short-term and long-term abiotic stress conditions playing a protective role against oxidative damage [87]. Finally, the increase in salicylic acid (a phytohormone and signaling metabolite) content, largely involved in increasing plant defenses against both abiotic and biotic stress [88], further confirmed that plants were able to activate several pathways necessary to cope with the stress induced by nerolidol.

## 5. Conclusions

The purpose of a natural herbicide is not to completely eradicate weeds, but to effectively reduce their fitness, providing greater competitiveness to the crops, and at the same time, assuring the maintenance of biodiversity within the agroecosystem. Therefore, the use of natural compounds at low dosages that are highly effective on weed metabolism and are easily degraded in the environment, could be a good choice for agricultural practices. Our results suggest that nerolidol reduced growth and development of *A. thaliana* and induced morphological changes in the root system by affecting the auxin balance, and inducing oxidative stress pathways. Moreover, at the concentration assayed, the *Arabidopsis* seedlings were able to counteract the stress by activating several biochemical and metabolic strategies aimed to increase the first enzymatic line of defense against ROS (increased activities of SOD and CAT), as well as the production of metabolites with osmoprotectant activity. All these effects, as well as the strategies adopted by the plant to cope with the stress, demand high energy consumption, and results in the strong inhibition of root growth and development, which renders nerolidol as a promising natural herbicidal compound for weed management.

## Supplementary Materials

The following supplementary materials are available online at https://www.mdpi.com/2223-7747/9/10/1347/s1, Table S1: Raw GC-MS based metabolomics data and output of the PCA analysis demonstrating the effects of nerolidol treatment (14 d) on *Arabidopsis thaliana* seedlings roots. Table S2: Data used in the generation of volcano plot for nerolidol treatment (14 d) on *A. thaliana* seedlings roots. Table S3: Results of the KEGG-based “pathway analysis” reporting all the data concerning the impact of nerolidol treatment (14 d) on *A. thaliana* seedlings roots metabolism.

## References

[1] Oerke E. (2006). Crop losses to pests. J. Agric. Sci..

[2] Moss S. (2019). Integrated weed management (IWM): Why are farmers reluctant to adopt non-chemical alternatives to herbicides?. Pest Manag. Sci..

[3] Tranel P.J., Wright T.R. (2002). Resistance of weeds to ALS-inhibiting herbicides: What have we learned?. Weed Sci..

[4] Pallett K. (1997). Herbicide target sites: Recent trends and new challenges. Proceedings of the Brighton Crop Protection Conference Weeds.

[5] Murphy B.P., Tranel P.J. (2019). Target-site mutations conferring herbicide resistance. Plants.

[6] Pallett K. (2016). Can we expect new herbicides with novel modes of action in the foreseeable future?. Outlooks Pest Manag..

[7] Cole D., Pallett K., Rodgers M. (2000). Discovering new modes of action for herbicides and the impact of genomics. Pestic. Outlook.

[8] Farooq N., Abbas T., Tanveer A., Jabran K. (2020). Allelopathy for weed management. Co-Evolution of Secondary Metabolites.

[9] Araniti F., Marrelli M., Lupini A., Mercati F., Statti G.A., Abenavoli M.R. (2014). Phytotoxic activity of *Cachrys pungens* Jan, a mediterranean species: Separation, identification and quantification of potential allelochemicals. Acta Physiol. Plant..

[10] Araniti F., Lupini A., Sorgonà A., Statti G.A., Abenavoli M.R. (2013). Phytotoxic activity of foliar volatiles and essential oils of *Calamintha nepeta* (L.) Savi. Nat. Prod. Res..

[11] Buhler D.D. (2002). 50th Anniversary—Invited Article: Challenges and opportunities for integrated weed management. Weed Sci..

[12] Scavo A., Mauromicale G. (2020). Integrated weed management in herbaceous field crops. Agronomy.

[13] Duke S.O., Owens D.K., Dayan F.E., Korres N.E., Burgos N.R., Duke S.O. (2018). Natural product-based chemical herbicides. Weed Control: Sustainability, Hazards, and Risks in Cropping Systems Worldwide.

[14] Araniti F., Grana E., Krasuska U., Bogatek R., Reigosa M.J., Abenavoli M.R., Sanchez-Moreiras A.M. (2016). Loss of gravitropism in farnesene-treated arabidopsis is due to microtubule malformations related to hormonal and ROS unbalance. PLoS ONE.

[15] Araniti F., Bruno L., Sunseri F., Pacenza M., Forgione I., Bitonti M.B., Abenavoli M.R. (2017). The allelochemical farnesene affects *Arabidopsis thaliana* root meristem altering auxin distribution. Plant Physiol. Biochem..

[16] Araniti F., Costas-Gil A., Cabeiras-Freijanes L., Lupini A., Sunseri F., Reigosa M.J., Abenavoli M.R., Sánchez-Moreiras A.M. (2018). Rosmarinic acid induces programmed cell death in Arabidopsis seedlings through reactive oxygen species and mitochondrial dysfunction. PLoS ONE.

[17] Araniti F., Miras-Moreno B., Lucini L., Landi M., Abenavoli M.R. (2020). Metabolomic, proteomic and physiological insights into the potential mode of action of thymol, a phytotoxic natural monoterpenoid phenol. Plant Physiol. Biochem..

[18] Duke S.O., Dayan F.E. (2015). Discovery of new herbicide modes of action with natural phytotoxins. Discovery and Synthesis of Crop Protection Products.

[19] Duke S.O., Romagni J.G., Dayan F.E. (2000). Natural products as sources for new mechanisms of herbicidal action. Crop Prot..

[20] Chan W.-K., Tan L.T.-H., Chan K.-G., Lee L.-H., Goh B.-H. (2016). Nerolidol: A sesquiterpene alcohol with multi-faceted pharmacological and biological activities. Molecules.

[21] Saito A.Y., Rodriguez A.A.M., Vega D.S.M., Sussmann R.A., Kimura E.A., Katzin A.M. (2016). Antimalarial activity of the terpene nerolidol. Int. J. Antimicrob. Agents.

[22] Ceole L.F., Cardoso M.D.G., Soares M.J. (2017). Nerolidol, the main constituent of *Piper aduncum* essential oil, has anti-*Leishmania braziliensis* activity. Parasitology.

[23] Araniti F., Sánchez-Moreiras A.M., Graña E., Reigosa M.J., Abenavoli M.R. (2017). Terpenoid *trans*-caryophyllene inhibits weed germination and induces plant water status alteration and oxidative damage in adult Arabidopsis. Plant Biol..

[24] Benvenuti S., Cioni P., Flamini G., Pardossi A. (2017). Weeds for weed control: Asteraceae essential oils as natural herbicides. Weed Res..

[25] Araniti F., Lupini A., Mauceri A., Zumbo A., Sunseri F., Abenavoli M.R. (2018). The allelochemical *trans*-cinnamic acid stimulates salicylic acid production and galactose pathway in maize leaves: A potential mechanism of stress tolerance. Plant Physiol. Biochem..

[26] Araniti F., Scognamiglio M., Chambery A., Russo R., Esposito A., D’Abrosca B., Fiorentino A., Lupini A., Sunseri F., Abenavoli M.R. (2017). Highlighting the effects of coumarin on adult plants of *Arabidopsis thaliana* (L.) Heynh. by an integrated-omic approach. J. Plant Physiol..

[27] Misra B.B., Das V., Landi M., Abenavoli M., Araniti F. (2020). Short-term effects of the allelochemical umbelliferone on *Triticum durum* L. metabolism through GC-MS based untargeted metabolomics. Plant Sci..

[28] Araniti F., Graña E., Reigosa M.J., Sánchez-Moreiras A.M., Abenavoli M.R. (2013). Individual and joint activity of terpenoids, isolated from *Calamintha nepeta* extract, on *Arabidopsis thaliana*. Nat. Prod. Res..

[29] Rawlinson C., Kamphuis L.G., Gummer J.P., Singh K.B., Trengove R.D. (2015). A rapid method for profiling of volatile and semi-volatile phytohormones using methyl chloroformate derivatisation and GC–MS. Metabolomics.

[30] Araniti F., Lupini A., Sunseri F., Abenavoli M.R. (2017). Allelopatic potential of *Dittrichia viscosa* (L.) W. Greuter mediated by VOCs: A physiological and metabolomic approach. PLoS ONE.

[31] Bradford M.M. (1976). A rapid and sensitive method for the quantitation of microgram quantities of protein utilizing the principle of protein-dye binding. Anal. Biochem..

[32] Tsugawa H., Cajka T., Kind T., Ma Y., Higgins B., Ikeda K., Kanazawa M., VanderGheynst J., Fiehn O., Arita M. (2015). MS-DIAL: Data-independent MS/MS deconvolution for comprehensive metabolome analysis. Nat. Methods.

[33] Kopka J., Schauer N., Krueger S., Birkemeyer C., Usadel B., Bergmüller E., Dörmann P., Weckwerth W., Gibon Y., Stitt M. (2005). GMD@ CSB. DB: The Golm metabolome database. Bioinformatics.

[34] Horai H., Arita M., Kanaya S., Nihei Y., Ikeda T., Suwa K., Ojima Y., Tanaka K., Tanaka S., Aoshima K. (2010). MassBank: A public repository for sharing mass spectral data for life sciences. J. Mass Spectrom..

[35] Sumner L.W., Amberg A., Barrett D., Beale M.H., Beger R., Daykin C.A., Fan T.W.-M., Fiehn O., Goodacre R., Griffin J.L. (2007). Proposed minimum reporting standards for chemical analysis. Metabolomics.

[36] Belz R.G., Hurle K., Duke S.O. (2005). Dose-response—A challenge for allelopathy?. Nonlinearity Biol. Toxicol. Med..

[37] Chong J., Soufan O., Li C., Caraus I., Li S., Bourque G., Wishart D.S., Xia J. (2018). MetaboAnalyst 4.0: Towards more transparent and integrative metabolomics analysis. Nucleic Acids Res..

[38] Reich M., Liefeld T., Gould J., Lerner J., Tamayo P., Mesirov J.P. (2006). GenePattern 2.0. Nat. Genet..

[39] Araniti F., Mancuso R., Lupini A., Giofrè S.V., Sunseri F., Gabriele B., Abenavoli M.R. (2015). Phytotoxic potential and biological activity of three synthetic coumarin derivatives as new natural-like herbicides. Molecules.

[40] Araniti F., Mancuso R., Lupini A., Sunseri F., Abenavoli M.R., Gabriele B. (2020). Benzofuran-2-acetic esters as a new class of natural-like herbicides. Pest Manag. Sci..

[41] Graña E., Sotelo T., Díaz-Tielas C., Araniti F., Krasuska U., Bogatek R., Reigosa M.J., Sánchez-Moreiras A.M. (2013). Citral induces auxin and ethylene-mediated malformations and arrests cell division in *Arabidopsis thaliana* roots. J. Chem. Ecol..

[42] Li X., Gruber M.Y., Hegedus D.D., Lydiate D.J., Gao M.-J. (2011). Effects of a coumarin derivative, 4-methylumbelliferone, on seed germination and seedling establishment in Arabidopsis. J. Chem. Ecol..

[43] Romero-Puertas M., McCarthy I., Gómez M., Sandalio L., Corpas F., Del Rio L., Palma J. (2004). Reactive oxygen species-mediated enzymatic systems involved in the oxidative action of 2, 4-dichlorophenoxyacetic acid. Plant Cell Environ..

[44] Liptáková L.u., Bočová B., Huttová J., Mistrík I., Tamás L. (2012). Superoxide production induced by short-term exposure of barley roots to cadmium, auxin, alloxan and sodium dodecyl sulfate. Plant Cell Rep..

[45] Causin H.F., Roqueiro G., Petrillo E., Láinez V., Pena L.B., Marchetti C.F., Gallego S.M., Maldonado S.I. (2012). The control of root growth by reactive oxygen species in *Salix nigra* Marsh. seedlings. Plant Sci..

[46] Dunand C., Crèvecoeur M., Penel C. (2007). Distribution of superoxide and hydrogen peroxide in Arabidopsis root and their influence on root development: Possible interaction with peroxidases. New Phytol..

[47] Pasternak T., Potters G., Caubergs R., Jansen M.A. (2005). Complementary interactions between oxidative stress and auxins control plant growth responses at plant, organ, and cellular level. J. Exp. Bot..

[48] Vladimirov Y.A., Olenev V., Suslova T., Cheremisina Z. (1980). Lipid peroxidation in mitochondrial membrane. Advances in Lipid Research.

[49] Arpagaus S., Rawyler A., Braendle R. (2002). Occurrence and characteristics of the mitochondrial permeability transition in plants. J. Biol. Chem..

[50] Huang T., Jander G. (2017). Abscisic acid-regulated protein degradation causes osmotic stress-induced accumulation of branched-chain amino acids in *Arabidopsis thaliana*. Planta.

[51] Araújo W.L., Tohge T., Ishizaki K., Leaver C.J., Fernie A.R. (2011). Protein degradation–an alternative respiratory substrate for stressed plants. Trends Plant Sci..

[52] Dat J., Vandenabeele S., Vranova E., Van Montagu M., Inzé D., Van Breusegem F. (2000). Dual action of the active oxygen species during plant stress responses. Cell. Mol. Life Sci. CMLS.

[53] Mittler R., Vanderauwera S., Gollery M., Van Breusegem F. (2004). Reactive oxygen gene network of plants. Trends Plant Sci..

[54] Mishra B.S., Singh M., Aggrawal P., Laxmi A. (2009). Glucose and auxin signaling interaction in controlling *Arabidopsis thaliana* seedlings root growth and development. PLoS ONE.

[55] Sairanen I., Novák O., Pěnčík A., Ikeda Y., Jones B., Sandberg G., Ljung K. (2012). Soluble carbohydrates regulate auxin biosynthesis via PIF proteins in Arabidopsis. Plant Cell.

[56] Dumont S., Rivoal J. (2019). Consequences of oxidative stress on plant glycolytic and respiratory metabolism. Front. Plant Sci..

[57] Rai V. (2002). Role of amino acids in plant responses to stresses. Biol. Plant..

[58] Podlešáková K., Ugena L., Spíchal L., Doležal K., De Diego N. (2019). Phytohormones and polyamines regulate plant stress responses by altering GABA pathway. New Biotechnol..

[59] Sami F., Yusuf M., Faizan M., Faraz A., Hayat S. (2016). Role of sugars under abiotic stress. Plant Physiol. Biochem..

[60] Salvi P., Kamble N.U., Majee M. (2018). Stress-inducible galactinol synthase of chickpea (CaGolS) is implicated in heat and oxidative stress tolerance through reducing stress-induced excessive reactive oxygen species accumulation. Plant Cell Physiol..

[61] Wójcik M., Skórzyńska-Polit E., Tukiendorf A. (2006). Organic acids accumulation and antioxidant enzyme activities in *Thlaspi caerulescens* under Zn and Cd stress. Plant Growth Regul..

[62] Sairam R., Tyagi A. (2004). Physiology and molecular biology of salinity stress tolerance in plants. Curr. Sci..

[63] Hare P.D., Cress W.A., Van Staden J. (1998). Dissecting the roles of osmolyte accumulation during stress. Plant Cell Environ..

[64] Sakamoto A., Murata N. (2002). The role of glycine betaine in the protection of plants from stress: Clues from transgenic plants. Plant Cell Environ..

[65] Zulfiqar F., Akram N.A., Ashraf M. (2020). Osmoprotection in plants under abiotic stresses: New insights into a classical phenomenon. Planta.

[66] Kumar N., Gautam A., Dubey A.K., Ranjan R., Pandey A., Kumari B., Singh G., Mandotra S., Chauhan P.S., Srikrishna S. (2019). GABA mediated reduction of arsenite toxicity in rice seedling through modulation of fatty acids, stress responsive amino acids and polyamines biosynthesis. Ecotoxicol. Environ. Saf..

[67] Fraire-Velázquez S., Balderas-Hernández V.E. (2013). Abiotic stress in plants and metabolic responses. Abiotic Stress—Plant Responses and Applications in Agriculture.

[68] Nishizawa A., Yabuta Y., Shigeoka S. (2008). Galactinol and raffinose constitute a novel function to protect plants from oxidative damage. Plant Physiol..

[69] Bouche N., Fromm H. (2004). GABA in plants: Just a metabolite?. Trends Plant Sci..

[70] Kinnersley A.M., Turano F.J. (2000). Gamma aminobutyric acid (GABA) and plant responses to stress. Crit. Rev. Plant Sci..

[71] Steward F.C., Thompson J.F., Dent C.E. (1949). γ-Aminobutyric acid: A constituent of the potato tuber?. Science.

[72] Fromm H. (2020). GABA signaling in plants: Targeting the missing pieces of the puzzle. J. Exp. Bot..

[73] Bor M., Turkan I. (2019). Is there a room for GABA in ROS and RNS signalling?. Environ. Exp. Bot..

[74] Seifikalhor M., Aliniaeifard S., Hassani B., Niknam V., Lastochkina O. (2019). Diverse role of γ-aminobutyric acid in dynamic plant cell responses. Plant Cell Rep..

[75] Good A.G., Zaplachinski S.T. (1994). The effects of drought stress on free amino acid accumulation and protein synthesis in *Brassica napus*. Physiol. Plant..

[76] Farhangi-Abriz S., Ghassemi-Golezani K. (2016). Improving amino acid composition of soybean under salt stress by salicylic acid and jasmonic acid. J. Appl. Bot. Food Qual..

[77] Yuan T.T., Xu H.H., Zhang K.X., Guo T.T., Lu Y.T. (2014). Glucose inhibits root meristem growth via ABA INSENSITIVE 5, which represses PIN1 accumulation and auxin activity in Arabidopsis. Plant Cell Environ..

[78] Mishra P., Dubey R. (2013). Excess nickel modulates activities of carbohydrate metabolizing enzymes and induces accumulation of sugars by upregulating acid invertase and sucrose synthase in rice seedlings. Biometals.

[79] Couée I., Sulmon C., Gouesbet G., El Amrani A. (2006). Involvement of soluble sugars in reactive oxygen species balance and responses to oxidative stress in plants. J. Exp. Bot..

[80] Bogdanović J., Mojović M., Milosavić N., Mitrović A., Vučinić Ž., Spasojević I. (2008). Role of fructose in the adaptation of plants to cold-induced oxidative stress. Eur. Biophys. J..

[81] Yang L., Zhao X., Zhu H., Paul M., Zu Y., Tang Z. (2014). Exogenous trehalose largely alleviates ionic unbalance, ROS burst, and PCD occurrence induced by high salinity in Arabidopsis seedlings. Front. Plant Sci..

[82] Chaves M.M., Pereira J.S., Maroco J., Rodrigues M.L., Ricardo C.P.P., Osório M.L., Carvalho I., Faria T., Pinheiro C. (2002). How plants cope with water stress in the field? Photosynthesis and growth. Ann. Bot..

[83] David M.M., Coelho D., Barrote I., Correia M.J. (1998). Leaf age effects on photosynthetic activity and sugar accumulation in droughted and rewatered *Lupinus albus* plants. Funct. Plant Biol..

[84] Chia D.W., Yoder T.J., Reiter W.-D., Gibson S.I. (2000). Fumaric acid: An overlooked form of fixed carbon in Arabidopsis and other plant species. Planta.

[85] Fernie A.R., Martinoia E. (2009). Malate. Jack of all trades or master of a few?. Phytochemistry.

[86] El-Tohamy W., El-Abagy H., Badr M., Gruda N. (2013). Drought tolerance and water status of bean plants (*Phaseolus vulgaris* L.) as affected by citric acid application. J. Appl. Bot. Food Qual..

[87] Minocha R., Majumdar R., Minocha S.C. (2014). Polyamines and abiotic stress in plants: A complex relationship1. Front. Plant Sci..

[88] Li Z., Yu J., Peng Y., Huang B. (2017). Metabolic pathways regulated by abscisic acid, salicylic acid and γ-aminobutyric acid in association with improved drought tolerance in creeping bentgrass (*Agrostis stolonifera*). Physiol. Plant..

